# Insecticide resistance and target site mutations (G119S *ace-1* and L1014F *kdr*) of *Culex pipiens* in Morocco

**DOI:** 10.1186/s13071-018-2625-y

**Published:** 2018-01-22

**Authors:** Fatim-Zohra Tmimi, Chafika Faraj, Meriem Bkhache, Khadija Mounaji, Anna-Bella Failloux, M’hammed Sarih

**Affiliations:** 1 Institut Pasteur du Maroc, Service de Parasitologie et des Maladies Vectorielles, Place Louis Pasteur, 20360 Casablanca, Morocco; 2 Faculté des Sciences Ain-Chock, Laboratoire de Physiopathologie, Génétique Moléculaire et Biotechnologie, Casablanca, Morocco; 3 Institut National d’Hygiène, Laboratoire d’Entomologie Médicale, Rabat, Morocco; 4 Institut Pasteur, Department of Virology, Arboviruses and Insect Vectors, 25-28 rue du Docteur Roux, 75724 Paris, France

**Keywords:** *Culex pipiens*, L1014F *kdr*, G119S *ace-1*, Insecticides, Resistance, Morocco

## Abstract

**Background:**

Control of the mosquito vector *Culex pipiens* with insecticides is the main way to control arboviruses that the species can transmit such as West Nile virus (WNV) and Rift Valley fever virus (RVFV). However, its efficiency has been hampered by the emergence of insecticide resistance. Little is known about the insecticide-resistance status and underlying resistance mechanisms of field-collected populations of *Cx. pipiens* in Morocco.

**Methods:**

Mosquito adults from Mohammadia city in Morocco were reared from immature stages. The level of their susceptibility to insecticides was assessed using standard WHO bioassay. The two forms of the *Cx. pipiens* complex and their hybrids were identified by a multiplex PCR. Identified mosquitoes were then tested for the presence of the G119S *ace-1* and L1014F *kdr* mutations using PCR-RFLP and PCR assays, respectively.

**Results:**

WHO bioassays indicated that *Cx. pipiens* was resistant to all tested insecticides: lambda-cyhalothrin (49% mortality), permethrin (63% mortality), DDT (16% mortality), malation (52% mortality) and bendiocarb (39% mortality). The frequency of the 119S allele was almost identical in the pipiens form and hybrids (0.11 and 0.15, respectively) whereas it remained low in the molestus form (0.03). No significant correlation was observed between the G119S allele and the resistance phenotype to two tested insecticides (malathion and bendiocarb). The frequency of the L1014F allele was identical in the pipiens form and hybrids (0.44) whereas it was low in the molestus form (0.36) but no significant difference was detected (*χ*^2^ *=* 1.46, *df* = 1, *P =* 0.225). The presence of the L1014F *kdr* mutation was significantly associated with resistance to three tested insecticides in pipiens form (*P* = 0.0019, *P* = 0.0023 and *P* = 0.023, respectively, to lambda-cyhalothrin, permethrin and DDT) whereas no significant correlation was observed between the L1014F *kdr* mutation and resistance phenotype in molestus form and hybrids to the three tested insecticides.

**Conclusion:**

These findings showed that wild populations of *Cx. pipiens* have developed resistance against the main insecticide families with different modes of action: organochlorines (DDT), organophosphates (malathion), carbamates (bendiocarb), pyrethroids (lambda-cyhalothrin, permethrin). Therefore, urgent action should be taken to manage the resistance in this species to maintain the effectiveness of arbovirus control.

## Background

The *Culex pipiens* complex mosquitoes are known to be competent vectors of West Nile virus (WNV) and Rift Valley fever virus (RVFV). WNV has been circulating for a very long time in the Mediterranean region [1–4]. In Morocco, several outbreaks of WNV have been reported. The first was in 1996; 94 equine cases including 42 deaths and only one human case was infected [5]. The second was in September and October of 2003 when WNV occurred among horses in Kenitra [6], and the last outbreak was in 2010 in Mohammadia region [7]. Unfortunately, there are no effective vaccines; the only way to limit the infection is the control of mosquitoes, by the most commonly used insecticides, namely organophosphates (temephos) for larvae and pyrethroids for adults. However, the overuse of these products causes the selection of resistance rendering the molecules ineffective for vector control [8].

There are different types of insecticide resistance mechanisms in mosquitoes, but the two main ones are (i) the metabolic resistance, grouping three major families of enzymes involved, glutathione S-transferase (GST), multifunctional monooxygenases (MFOs) and carboxyl-esterase (COE), and (ii) the target site modification, which is due to point mutations in the target of insecticides, thus limiting the binding of the neurotoxin products. Several insecticide target modifications have been described: the γ-amino butyric acid (GABA Receptors) encoded by *Rdl* gene, the synaptic acethylcholinesterase (AChE1) encoded by the *ace-1* gene and the voltage-dependent sodium channel encoded by the *kdr* gene [9, 10]. The knockdown resistance *kdr* gene is the major mechanism responsible for resistance to DDT and PYR, reducing the sensitivity of the receptors to these products in the voltage-gated sodium channel (VGSC) across the neural axon [8, 11]. This resistance is due to the mutations of *kdr* gene. Many mutations have been reported, but the most common known to be associated with knockdown resistance in mosquitoes, including *Cx. pipiens*, are L1014F by the substitution of a leucine (TTA) by phenylalanine (TTT) and L1014S by the leucine (TTA) to serine (TCA) substitution at codon 1014 [12–14], while the L1014C mutation by the substitution of a leucine (TTA) by cysteine (TGT) has only been reported for *Cx. pipiens molestus* from China [15].

The enzyme AChE is the target of OP and CX, which are competitive inhibitors of acethylcholine (Ach). After binding to AChE, the insecticides prevent the hydrolysis of the neurotransmitter Ach in the cholinergic synapses of the central nervous system. As a result, the Ach remains active, and the nervous influx is continued, causing the death of the insect by tetany [16]. In several insects, two genes are described, *ace-1* and *ace-2*, coding for the two synaptic enzymes, AChE1 and AChE2, respectively. Five mutations were described in OP-resistant insects [17]. In mosquitoes, including *Cx. pipiens*, the most common resistance mutation is G119S in the *ace-1* gene [18], located near the catalytic site. The high insensitivity displayed by *Cx. pipiens* is due to the substitution of glycine by serine, resulting from a single point mutation GGC to AGC in *ace-1* gene [18], allowing a decreased inhibition of the main synaptic enzyme AChE1 by the insecticide [19].

In Morocco, research on insecticide resistance and the mechanisms responsible for insecticide resistance in *Cx. pipiens* remain incomplete. The only published studies are those on the resistance level of *Cx. pipiens* larvae to temephos [20, 21] and recently, our team described the presence of *kdr* mutation in different forms of *Cx. pipiens* [22]. This study aimed to evaluate the insecticide susceptibility status and investigate the target site mutation frequencies (G119S and L1014F) in *Cx. pipiens* (forms pipiens and molestus, and hybrids) from Mohammadia area that was affected by the last outbreaks of WNV.

## Methods

### Mosquito collection

Mosquitoes were collected as larvae using the dipping sampling method during summer 2016, from a shantytown in Mohammadia (33°34′91″N, 7°37′56″E). The site is a suburban habitat with aboveground breeding sites treated with insecticides. The larvae were reared to adults in the laboratory at 28 ± 1 °C with a relative humidity of 80% and a 16:8 h photoperiod. Mosquitoes were identified as *Cx. pipiens* using the dichotomous software for the identification of mosquitoes in Mediterranean Africa [23].

### Bioassays

Adult bioassays were carried out using WHO protocols, using four sets of 20–25 unfed and 2–5-day-old females. They were exposed to a filter paper impregnated with malathion (OP) at a dose of 5%, bendiocarb 0.1% (CX), DDT 4% (OC), lambda-cyalothrin 0.05% and permethrin 0.75% (PYR), corresponding to recommended concentrations to kill 100% of the susceptible individuals [24]. As a control, two sets of 20 unfed 2–5-day-old females were exposed to insecticide-free papers in test tubes. After exposure, mosquitoes were maintained at 28 ± 1 °C and 80 ± 10% relative humidity, with sugar solution provided. The knockdown effect (KD) was evaluated, at intervals of 10 min, for each test tube impregnated with PYR and DDT. The mortality was recorded 24 h after exposure. Dead and surviving individuals were frozen at -20 °C for molecular analysis.

### Molecular identification of *Cx. pipiens* forms

Mosquito DNA was extracted individually using the method of DNAzol according to the manufacturer’s protocol. Multiplex PCR assays were used to identify *Cx. pipiens* complex, described by Banck &Fonseca [25]. The CQ11 locus was used to distinguish between the two forms of *Cx. pipiens* (pipiens and molestus) and their hybrids. The DNA fragment size amplified varied between pipiens form (200 bp) and molestus form (250 bp), allowing us to distinguish the two forms in a single PCR reaction.

### Detection of G119S mutation

The presence of G119S was confirmed using diagnostic PCR-RFLP tests described by Weill et al. [26] (30 cycles of 94 °C for 30 s, 52 °C for 30 s and 72 °C for 1 min). PCR products were digested by *Alu*I, restriction enzyme, according to the manufacturer’s instructions (Jena Bioscience, Jena, Germany). After digestion, DNA fragments were separated by electrophoresis on 2% agarose gel and were visualized by ethidium bromide staining under ultraviolet light. Two fragments (74 bp and 120 bp) were obtained for homozygous resistant (RR) mosquitoes. The homozygous susceptible (SS) mosquitoes presented one undigested fragment of 194 bp while the heterozygous resistant (RS) individuals displayed a combined pattern (three fragments: 194, 120 and 74 bp).

### Detection of L1014F mutation

The presence of L1014F was investigated using two separate PCRs in parallel, one for wild-type susceptible allele detection, using the primers Cgd1, Cgd2 and Cgd3 and the other for leucine-phenylalanine substitution, by replacing Cgd3 by Cgd4. The PCR conditions were 1 min at 94 °C, 2 min at 48 °C and 2 min at 72 °C for 40 cycles, as described by Martinez Torrez et al. [12], which allow detecting only one form of resistance allele (1014F). Then the DNA fragments were separated by electrophoresis on 1.5% agarose gel and were visualized by ethidium bromide staining under ultraviolet light.

### Data analysis

The knockdown time for 50% (the median knockdown time ‘kdT50’) and 90% of exposed mosquitoes to PYR and DDT were estimated using a log time probit model (WINDL software). OP and CX do not induce any knockdown effect. The odds ratio (OR) test was applied to estimate the association between *ace-1*-resistant allele 119S and the resistant phenotype and the association between *kdr*-resistant allele 1014F and resistant phenotype to different insecticides.

## Results

### Insecticide susceptibility bioassays

*Culex pipiens* mosquitoes from the study area showed resistance to all tested insecticides (Table 1). For organophosphates (malathion) and carbamates (bendiocarb), resistance was observed in mosquitoes with mortality rates of 52% and 39%, respectively. While for pyrethroids, resistance was observed in mosquitoes with mortality rates of 49% and 63%, respectively, for lambda-cyalothrin and permethrin. In contrast, DDT caused only 16% of mortality, which revealed a low susceptibility. KDT_50_ and KDT_90_ were 33 min and 61 min, respectively, for lambda-cyalothrin, and 33 min and 65 min, respectively, for permethrin (Table 1).

**Table 1:** Mortality and knockdown effect of insecticides on *Cx. pipiens* in Mohammadia, Morocco

Insecticide	Exposed mosquitoes		Control mosquitoes		kdT_50_ (min) (95% CI)	kdT_90_ (min) (95% CI)
	*n*	Mortality (%)	*n*	Mortality (%)		
Lymbda-cyhalothrin (0.05%)	111	49	40	0	33 (30.52–34.95)	61 (56.23–69.20)
Permethrin (0.75%)	118	63	40	0	33 (30.07–35.07)	65 (59.09–74.43)
DDT (4%)	110	16	40	0	no kd	no kd
Malathion (5%)	90	52	40	0	–	–
Bendiocarb (0.1%)	102	39	40	0	–	–

*Abbreviations*: *kdT*_*50*_ and *kdT*_*90*_ knock down time for 50% and 90%, respectively, of exposed mosquitoes with confidence intervals (CI) at 5% level provided by WINDL software, *n* number of mosquitoes

### Frequencies of *Culex pipiens* forms

After insecticide bioassays, 531 insecticide-resistant and insecticide-susceptible mosquitoes were tested by PCR to identify the *Cx. pipiens* forms and hybrids. The highest frequency of insecticide-resistant *Cx. pipiens* was observed for the pipiens form with 67%, 63%, 49%, 48% and 67%, respectively, for malathion, bendiocarb, lambda-cyhalothtrin, permethrin and DDT. Hybrids represented 23%, 24%, 35%, 48% and 30%, respectively, while the molestus form represented only 10%, 13%, 16%, 5% and 2%, respectively (Tables 2 and 3).

**Table 2:** Frequencies of *ace-1* mutation according to the phenotype (resistant/susceptible) of different forms of *Cx. pipiens* in Mohammadia, Morocco

Insecticide	*Cx. pipiens* form	Phenotype	*n* (%)	Insecticide resistance (%)	Genotype (%)			Frequency of allele
					SS	RS	RR	
					*n* (%)	*n* (%)	*n* (%)	
Malathion	*Cx. p. pipiens*	Susceptible	30 (46.0)		22 (73.0)	8 (27.0)	0	0.13
		Resistant	35 (54.0)	67	25 (71.5)	10 (28.5)	0	0.14
		Total	65		47 (72.0)	18 (28.0)	0	0.14
	Hybrid	Susceptible	8 (40.0)		5 (62.5)	3 (37.5)	0	0.19
		Resistant	12 (60.0)	23	6 (50.0)	6 (50.0)	0	0.25
		Total	20		11 (55.0)	9 (45.0)	0	0.22
	*Cx. p. molestus*	Susceptible	2 (28.5)		2 (100)	0	0	0
		Resistant	5 (71.5)	10	4 (80.0)	1 (20.0)	0	0.10
		Total	7		6 (86.0)	1 (14.0)	0	0.07
Bendiocarb	*Cx. p. pipiens*	Susceptible	23 (37.0)		22 (96.0)	1 (4.0)	0	0.02
		Resistant	39 (63.0)	63	31 (79.5)	8 (20.5)	0	0.10
		Total	62		53 (85.0)	9 (15.0)	0	0.07
	Hybrid	Susceptible	16 (52.0)		15 (94.0)	1 (6.0)	0	0.03
		Resistant	15 (48.0)	24	10 (67.0)	5 (33.0)	0	0.17
		Total	31		25 (81.0)	6 (19.0)	0	0.09
	*Cx. p. molestus*	Susceptible	1 (11.0)		1 (100)	0	0	0
		Resistant	8 (89.0)	13	8 (100)	0	0	0
		Total	9		9 (100)	0	0	0

*Abbreviations*: *RR* homozygote resistant, *RS* heterozygote resistant, *SS* homozygote susceptible

**Table 3:** Frequencies of *kdr* mutation according to the phenotype (resistant/susceptible) of different forms of *Cx. pipiens* in Mohammadia, Morocco

Insecticide	*Cx. pipiens* form	Phenotype	*n* (%)	Insecticide resistance (%)	Genotype			Frequency of allele
					SS *n* (%)	RS *n* (%)	RR *n* (%)	
Lambda-cyhalothrin	*Cx. p. pipiens*	Susceptible	23 (45.0)		14 (61.0)	8 (35.0)	1 (4.0)	0.39
		Resistant	28 (55.0)	49	4 (14.0)	19 (68.0)	5 (18.0)	0.52
		Total	51		18 (35.0)	27 (53.0)	6 (12.0)	0.38
	Hybrid	Susceptible	25 (56.0)		17 (68.0)	6 (24.0)	2 (8.0)	0.20
		Resistant	20 (44.0)	35	2 (10.0)	16 (80.0)	2 (10.0)	0.50
		Total	45		19 (42.0)	22 (49.0)	5 (11.0)	0.35
	*Cx. p. molestus*	Susceptible	6 (40.0)		3 (50.0)	3 (50.0)	0	0.25
		Resistant	9 (60.0)	16	2 (22.0)	7 (78.0)	0	0.39
		Total	15		5 (33.0)	10 (67.0)	0	0.33
Permethrin	*Cx. p. pipiens*	Susceptible	28 (57.0)		9 (32.0)	19 (68.0)	0	0.34
		Resistant	21 (43.0)	48	3 (14.0)	14 (67.0)	4 (19.0)	0.52
		Total	49		12 (24.5)	33 (67.0)	4 (8.0)	0.42
	Hybrid	Susceptible	37 (64.0)		7 (19.0)	28 (76.0)	2 (5.0)	0.43
		Resistant	21 (36.0)	48	3 (14.0)	15 (72.0)	3 (14.0)	0.50
		Total	58		10 (17.0)	43 (74.0)	5 (9.0)	0.45
	*Cx. p. molestus*	Susceptible	9 (82.0)		3 (33.0)	6 (67.0)	0	0.33
		Resistant	2 (18.0)	5	1 (50.0)	1 (50.0)	0	0.25
		Total	11		4 (36.0)	7 (64.0)	0	0.32
DDT	*Cx. p. pipiens*	Susceptible	10 (14.0)		8 (80.0)	2 (20.0)	0	0.10
		Resistant	62 (86.0)	67	10 (16.0)	44 (71.0)	8 (13.0)	0.48
		Total	72		18 (25.0)	46 (64.0)	8 (11.0)	0.43
	Hybrid	Susceptible	4 (12.5)		2 (50.0)	2 (50.0)	0	0.25
		Resistant	28 (87.5)	30	2 (7.0)	20 (71.5)	6 (21.5)	0.57
		Total	32		4 (12.0)	22 (69.0)	6 (19.0)	0.53
	*Cx. p. molestus*	Susceptible	4 (67.0)		0	4 (100)	0	0.50
		Resistant	2 (33.0)	2	0	2 (100)	0	0.50
		Total	6		0	6 (100)	0	0.50

*Abbreviations*: *RR* homozygote resistant, *RS* heterozygote resistant,*SS* homozygote susceptible

### Genotyping of target-site mutations: *ace-1* and *kdr*

A total of 531 female mosquitoes, exposed to the five neurotoxin products, were analyzed for the G119S *ace-1* and L1014F *kdr* mutations.

The mosquitoes used for the bioassays with malathion (92 specimens) and bendiocarb (102 specimens) were genotyped for the G119S mutation. The frequency of genotypes is presented in Table 2. When treated with malathion and bendiocard, no homozygote resistant genotypes were detected. Most mosquitoes presented a SS or RS genotype. In addition, 339 mosquitoes were analyzed for the L1014F *kdr* mutation (Table 3). After exposure to lambda-cyalothrin, most resistant mosquitoes had a RS genotype (68%, 80% and 78% for the pipiens, hybrid and molestus forms, respectively) and susceptible mosquitoes presented a SS genotype (61%, 68% and 50% for the pipiens, hybrid and molestus forms, respectively). Only few individuals had RR genotypes.

With permethrin, most resistant mosquitoes were scored as a RS genotype: 67% for the pipiens form, 72% for the hybrid form and 50% for the molestus form. Surprisingly, a high proportion of individuals with susceptible phenotype were scored as a RS genotype: 68% for the pipiens form, 76% for the hybrid form and 67% for the molestus form. Only few individuals presented a RR genotype.

After exposure to DDT, only a low proportion of mosquitoes had a RR genotype: 13% for the pipiens form and 21.5% for the hybrid form. Most DDT-resistant mosquitoes had a RS genotype: 71% for the pipiens from, 71.5% for the hybrid form and 100% for the molestus form. Susceptible mosquitoes presented mostly a SS genotype: 80% for the pipiens form and 50% for the hybrid form.

The frequencies of the 119S allele and the 1014F allele are shown in Tables 2 and 3. The frequency of the 119S allele was almost identical in the pipiens form and hybrids, 0.11 and 0.15, respectively, whereas it was low in the molestus form (0.03). The frequency of the 1014F allele was identical in the pipiens and hybrid forms (0.44) whereas it was low in the molestus form (0.36) but no significant difference was detected (*χ*^2^ *=* 1.46, *df* = 1, *P* = 0.225*)*.

The diagnostic test using *ace-1* as a resistance marker in *Cx. pipiens* differed between malathion and bendiocarb, with a high specificity for both (72% and 95%, respectively) but markedly lower sensitivity (33% and 21%, respectively) (Table 4). The *kdr* as a resistance marker in *Cx. pipiens* differed between the three insecticides, with a high sensitivity (86 for lambda-cyhalothrin, 84 for permethrin and 87 for DDT) but markedly a lower specificity (63, 26, and 56, respectively) (Table 4).

**Table 4:** Diagnostic value of *ace-1* (malathion, bendiocarb) and *kdr* (lambda-cyhalothrin, permethrin, DTT) alleles for detection of resistance in *Cx. pipiens*

	Malathion	Bendiocarb	Lambda-cyhalothrin	Permethrin	DDT
Sensitivity, % (95% CI)	33 (20.33–47.11)	21 (11.66–33.18)	86 (74.21–93.74)	84 (69.93–93.36)	87 (73.74–95.06)
Specificity, % (95% CI)	72 (56.11–85.40)	95 (83.08–99.39)	63 (48.74–75.71)	26 (16.22–37.16)	56 (21.20–86.30)
PPV, % (95% CI)	61 (44.98–74.50)	87 (60.76–96.47)	71 (63.01–77.89)	40 (35.85–44.75)	91 (82.69–95.44)
NPV, % (95% CI)	45 (38.77–52.02)	44 (40.11–47.31)	81 (68.41–89.29)	73 (55.39–85.58)	45 (24.41–68.25)

*Abbreviations*: *CI* confidence interval, *PPV* positive predictive values, *NPV* negative predictive values

No significant association was shown between the G119S allele and the resistance phenotype to two tested insecticides (malathion and bendiocarb) (Table 5). The presence of the L1014F *kdr* mutation was significantly associated with resistance to three tested insecticides in the pipiens form (*P* = 0.0019, *P* = 0.0023 and *P* = 0.023, respectively, to lambda-cyhalothrin, permethrin and DDT). There was no significant correlation between the L1014F *kdr* mutation and resistance phenotype in the molestus and hybrid forms to three tested insecticides (Table 6).

**Table 5:** Correlation between the frequency of 119S allele and insecticide resistant/susceptible phenotypes to malathion and bendiocarb

Insecticide	*Cx. pipiens* form	Phenotype	*n*	G119S alleles		Odds ratio	*P*-value
				119G (S)	119S (R)		
Malathion	*Cx. p. pipiens*	Susceptible	30	52	8	1.08	0.875
		Resistant	35	60	10	0.40–2.95	
	Hybrid	Susceptible	8	13	3	1.44	0.464
		Resistant	12	18	6	0.30–6.87	
	*Cx. p. molestus*	Susceptible	2	4	0	1.42	0.511
		Resistant	5	9	1	0.05–42.25	
Bendiocarb	*Cx. p. pipiens*	Susceptible	23	45	1	5.14	0.093
		Resistant	39	70	8	0.62–42.54	
	Hybrid	Susceptible	16	31	1	6.20	0.071
		Resistant	15	25	5	0.68–56.59	
	*Cx. p. molestus*	Susceptible	1	2	0	na	
		Resistant	8	16	0	na	

*Abbreviation*: na, not applicable

**Table 6:** Correlation between the frequency of 1014F allele and insecticide resistant/susceptible phenotypes to lambda-cyhalothrin, permethrin and DDT

Insecticide	*Cx. pipiens* form	Phenotype	*n*	L1014F alleles		Odds ratio	*P*-value
				1014 L (S)	1014F (R)		
Lambda-cyhalothrin	*Cx. p. pipiens*	Susceptible	23	36	10	3.87	0.0019
		Resistant	28	27	29	1.61–9.28	
	Hybrid	Susceptible	25	40	10	4.00	0.070
		Resistant	20	20	20	1.58–10.14	
	*Cx. p. molestus*	Susceptible	6	9	3	1;91	0.429
		Resistant	9	11	7	3.38–9.59	
Permethrin	*Cx. p. pipiens*	Susceptible	28	37	19	2.14	0.0023
		Resistant	21	20	22	0.94–4.86	
	Hybrid	Susceptible	37	42	32	0.977	0.429
		Resistant	21	27	21	4.47–2.03	
	*Cx. p. molestus*	Susceptible	9	12	6	1.50	0.746
		Resistant	2	3	1	0.13–17.68	
DDT	*Cx. p. pipiens*	Susceptible	5	9	1	8.44	0.023
		Resistant	31	32	30	1.00–70.70	
	Hybrid	Susceptible	2	3	1	4.00	0.223
		Resistant	14	12	16	0.37–43.40	
	*Cx. p. molestus*	Susceptible	2	2	2	1.00	1
		Resistant	1	1	1	0.03–29.83	

## Discussion

*Culex pipiens* is an important vector of several diseases including West Nile fever. The lack of an effective vaccine drives the use of insecticides as the main way to control vector populations [27, 28]. In this study, we investigated the status of resistance of the *Cx. pipiens* complex to the most commonly used insecticides for vector control. We showed that *Cx. pipiens* mosquitoes of the study area presented high resistance levels to PYR (λ-cyalothrin and permethrin), OC (DDT), OP (malathion) and CX (bendiocarb). For PYR and DDT, the insecticide with the highest resistance, as shown by death rates, were DDT (16%), followed by λ-cyalothrin (46%) and permethrin (63%). Understanding the mechanisms underlying this resistance is essential to guide the use of these chemicals and preserve their efficacy as vector control tools. We also investigated the insecticide target site mutations: G119S *ace-1* and L1014F *kdr*. It has been reported that the *kdr* mutation was closely associated with PYR and DDT resistance in mosquitoes [13, 14, 29–32]. We found that the diagnostic values of the *ace-1* and *kdr* mutations are different: sensitivity is high for the *kdr* mutation (84–87%) compared to the *ace-1* mutation (21% and 33%). On the other hand, the specificity is higher for the *ace-1* mutation (72% and 95%) compared to the *kdr* mutation (26%, 56%, 63%).

The leucine (L) to phenylalanine (F) substitution at position 1014 was detected in *Cx. pipiens* mosquitoes [12]. This study showed that the distribution of the L1014F *kdr* mutation is widespread, particularly in the pipiens form and hybrids. Our previous report has documented the presence of a 1014 L/1014F genotype in three regions of Morocco including Tangier, Casablanca and Marrakech [22]. These results confirmed that the frequencies of 1014 L/1014F genotype are variable depending on the area [31, 32], and the L1014F *kdr* mutation is widespread in different sites of Morocco from the North (Tangier) to Marrakech in the South. However, many tested mosquitoes, presenting a resistant phenotype, were also susceptible for the *kdr* allele 1014F; this result suggests that *kdr* might not be the only mechanism conferring resistance to PYRs and DDT [33], but other mechanisms can be involved such an overproduction of detoxifying enzymes [34]. Our results are in agreement with those found by other teams [32, 35].

Other mutations were found in *Cx. pipiens*; the mutation from leucine to serine (TTA to TCA) had been reported in *Cx. p. quinquefasciatus* [13, 36, 37] and was also found in *Cx. pipiens* complex from China (frequencies ranging between 2.4–28.6%) [38], *Cx. p. pipiens* from China and the USA, *Cx. p. quinquefasciatus* from the USA and in *Cx. pipiens pallens* from Japan and China [15]. The L1014C mutation by the substitution of leucine (TTA) to cysteine (TGT) has been reported in many *Anopheles* species [39–41] and for *Cx. P. molestus* from China [15].

Mosquitoes with the *Phe/kdr* mutation displayed a high level of resistance to both pyrethroids and DDT, whereas those with the *Ser/kdr* mutation displayed a high level of resistance to DDT but a low level of resistance to pyrethroids [12, 42].

OP and CX are widely used around the world for vector control. The results of this study showed that all forms of the population treated with malathion and bendiocarb exhibit a high level of resistance. G119S is responsible for the reduction of AChE1 activity in cholinergic synapses. It is one of the most common mutations detected in *Cx. pipiens* mosquitoes [18]. The results showed that G119S mutation was present in the tested population presenting resistant phenotypes. In Morocco in 2002, Faraj et al. [21] found that larvae of *Cx. pipiens* developed varying degrees of resistance in four different provinces for certain organophosphates (temephos, chloropyriphos, fenitrothion, pirimiphos-methyl); the highest rates (> 250) were recorded in Mohammedia and the lowest in Salé (< 7). These results confirmed the presence of significant resistance in *Cx. pipiens* for chlorpyriphos and temephos in the prefectures of Mohammedia, Rabat and Skhirat-Témara. Resistance was also important in pyrimiphos-methyl in Mohammedia and malathion in Rabat and Skhirat Témara. In fact, temephos, chlorpyriphos, and pyrimiphos-methyl are widely used in the control of mosquito larvae in these areas, which may explain the high resistance levels to these products. Malathion, on the other hand, has not been reported among the products used in mosquito control by the counties of Rabat. Moreover, by comparing the sensitivity of the different populations of *Cx. pipiens* collected from these areas, it was found that this species developed resistance levels that vary according to the prefectures.

Also, the results found by El Ouali Lalami et al. [20], can be explained by the fact that chlorpyriphos and temephos are insecticides widely used in the control of mosquito larvae in the study area of Fez. The highest resistance level (14.34) to temephos was obtained in Sidi Hrazem, and the lowest (12.17) was recorded in the Hafat Moulay Driss. The resistance rates of the *Cx. pipiens* species to temephos recorded in the city of Fez were higher than the rates recorded in the prefecture of Sale but lower than those recorded in the prefectures of Temara, Rabat and Mohammedia. These observations are consistent with those reported in Tunisia by Kooli & Rhaiem [43], who reported that a high level of resistance was acquired in urban larval populations of *Cx. pipiens* after several treatments with organophosphates. Other authors [44] also reported high levels of resistance of *Cx. pipiens* larvae to temephos and fenthion (resistance ratios 129.23 and 115.56, respectively).

The resistance levels observed, if not due to intensive prior use, can only be explained by the acquisition of cross-resistance. Indeed, Sinegre et al. [45] found in *Cx. pipiens* treated with chlorpyriphos, the appearance of resistance to other organophosphates. Chavasse &Yap [46] were able to confirm that the prolonged use of an organophosphorus always leads to the appearance of cross-resistance to other organophosphates and sometimes to certain products of the carbamate family. Indeed, Sinegre et al. [47] were able to establish an obvious correlation between the degrees of resistance and the frequency of insecticide treatments. On the French Mediterranean coast, for example, a resistance rate of 60 was reached after seven years of regular control of *Cx. pipiens* larvae with chlorpyriphos [48]. According to the same author, it increased until a resistance rate of 330 in some areas after ten years of use. The high rates of resistance found in our study could be explained by the appearance of cross-resistance, especially when the area was treated only with temephos for larvae and PYR for adults.

Our results showed no correlation between the resistant phenotype and the presence of the G119S mutation. This result can be explained by the implication of metabolic mechanisms with overproduction of detoxifying enzymes. Target site modification coupled with enzyme detoxification has been described in *An. gambiae* and *Cx. quinquefasciatus* from Benin [49]. Other studies have demonstrated the likely implication of metabolic mechanisms in resistance to bendiocarb [50]. There is also a need for a future study to investigate metabolic resistance like glutathione S-transferase, carboxylesterase, and cytochrome P450 monooxygenase activity to elucidate the mechanisms of resistance to the currently used insecticides in vector control.

## Conclusion

The use of insecticides for vector control had been achieved in Morocco for a long time. The results of bioassays and molecular identification of target-site mutations showed clearly that *Cx. pipiens* mosquitoes from Mohammadia were resistant to all tested insecticides. The frequencies of resistant *ace-1* and *kdr* alleles carrying the G119S and L1014F substitution are dramatically high for *Cx. pipiens* populations collected in the study site. Hence it is pivotal that policy makers and program implementers recognize the growing threat posed by insecticide resistance and strive to integrate resistance management into all control programs.

## Abbreviations

AChE1: Acetylcholinesterase-1 enzyme
Cx: Carbamates
DDT: Dichlorodiphenyltrichloroethane
GABA: Gamma-amino butyric acid
Kd: Knockdown
*kdr*: Knockdown resistance
OP: Organophosphates
OR: Odds ratio
PYR: Pyrethroids
RVFV: Rift Valley fever virus
ULV: Ultra-low volume
WHO: World Health Organization
WNV: West Nile virus
